# Species complexes and phylogenetic lineages of *Hoferellus* (Myxozoa, Cnidaria) including revision of the genus: A problematic case for taxonomy

**DOI:** 10.1186/s13071-015-1265-8

**Published:** 2016-01-11

**Authors:** Gema Alama-Bermejo, Miloslav Jirků, Alena Kodádková, Hana Pecková, Ivan Fiala, Astrid S. Holzer

**Affiliations:** Institute of Parasitology, Biology Centre of the Czech Academy of Sciences, České Budějovice, 37005 Czech Republic; Marine Zoology Unit, Cavanilles Institute of Biodiversity and Evolutionary Biology, Science Park, University of Valencia, Paterna, 46980 Spain; Department of Microbiology, Oregon State University, Corvallis, OR 97331 USA

**Keywords:** Amphibia, Anura, fish, *Carassius*, *Cyprinus carpio*, ITS cloning, Mode of attachment, Teleostei, Ultrastructure

## Abstract

**Background:**

Myxozoans are metazoan parasites whose traditional spore morphology-based taxonomy conflicts DNA based phylogenies. Freshwater species of the genus *Hoferellus* are parasites of the excretory system, with several members infecting food and ornamental fish species, as well as amphibians. This study aims to increase our understanding of their molecular diversity and development, aspects about which little is known, and to generate a molecular diagnostic tool to discriminate between different pathogenic and non-pathogenic *Hoferellus* spp.

**Methods:**

SSU and ITS rDNA phylogeny, along with morphological descriptions using light and electron microscopy were used to identify and characterize *Hoferellus* species collected from the urinary system of fishes and frogs. A PCR-based diagnostic assay was designed to differentiate between cryptic *Hoferellus* spp in cyprinid fishes commonly cultured in Central Europe.

**Results:**

Our phylogenetic results separate the species of *Hoferellus* into two phylogenetic sublineages which are indistinguishable on the basis of generic morphological traits: 1) The *Hoferellus sensu stricto* sublineage, which is composed of the type species *Hoferellus cyprini*, *Hoferellus carassii* and a cryptic species, *Hoferellus* sp. detected only molecularly in common carp. 2) The *Hoferellus sensu lato* sublineage into which the new species we described in this study, *Hoferellus gnathonemi* sp. n. from the kidney of the elephantnose fish and *Hoferellus anurae* from reed frogs, are placed together with *Hoferellus gilsoni* previously sequenced from European eel. Apart from phylogenetic analyses, we also provide novel ultrastructural data on the phagocytotic nature of some *Hoferellus* plasmodia and on the elusive intracellular stages ascribed to the presporogonic development of this genus.

**Conclusions:**

We provide molecular evidence of the polyphyly of the genus *Hoferellus* and provide novel morphological details of its members. Based on the presented data, we revise and propose emendation of the genus *Hoferellus*.

## Background

Myxozoans are microscopic cnidarian parasites in aquatic environments and are known for the diseases they provoke in fisheries and aquaculture. They have complex life cycles alternating between intermediate vertebrate host, usually fish but also other vertebrates, and a definitive invertebrate host, annelids and bryozoans. Myxozoa have notoriously conflicting traditional morphology- and more recent phylogeny-based systematics. As a result, a paraphyletic and/or polyphyletic nature has been revealed in many myxozoan genera subjected to extensive sampling and subsequent phylogenetic analyses [1–4].

The urinary system of the vertebrate host is a common habitat for proliferation and spore formation of diverse myxozoan species [5, 6]. The common myxozoan ancestor was proposed to infect renal tubules in freshwater fish, a habitat that has been independently colonized by myxozoans several times during evolution [7]. Members of the genus *Hoferellus* Berg, 1898; syn. *Hoferia* Doflein, 1898 (hom. *Hoferia* Bittner, 1894), syn. *Mitraspora* Fujita, 1912 are parasites of the excretory system, including 25 nominal species infecting fish and one species infecting frogs (see review [5]). Myxospores of *Hoferellus* are miter-like or round with characteristic posterior filaments or a brush border at the posterior end. Based solely on morphological data, *Hoferellus* has suffered abundant taxonomic reassignments [8–13]. The type species, *Hoferellus cyprini* (Doflein, 1898) affects the renal system of common carp *Cyprinus carpio* L., one of the most cultured freshwater fish worldwide [14]. The integrity of *H. cyprini* as a species has been discussed due to elusive intracellular stages in the epithelium of the renal tubules and the difficulty of detecting spores [15, 16]. The most studied species, *Hoferellus carassii* Achmerov, 1960, originally described in gibel carp *Carassius gibelio* (Bloch, 1782), was identified as an agent of kidney enlargement disease (KED) in goldfish, *Carassius auratus* (L.), perhaps globally the most widespread ornamental fish, and later related to kidney bloater in farmed *C. auratus*, causing a polycystic, swollen kidney and consequent abdominal distension [17–21]. The identity, host specificity and putative occurrence of mixed infections of *H. carassii* and *H. cyprini* has been largely discussed and remains a puzzle [9, 12, 17, 19, 22–26]. Most other species are known solely based on morphological description of spores, and only scarce molecular information is available for the members of this genus (2 partial SSU rDNA sequences).

An annual life cycle was assigned to *H. carassii* and *H. cyprini* [17, 20, 27, 28]. The developmental cycle includes intracellular stages in the epithelium of the renal tubules [20, 27] that were not detected by all authors [25] or ascribed to another myxozoan genus, *Sphaerospora*, which is expected to be unable to complete its development in the kidney [16, 29]. Luminal stages of *Hoferellus gilsoni* (Debaisieux, 1925) are characterized by a mode of attachment in the urinary bladder of *Anguilla anguilla* (L.), which is unique amongst Myxozoa, including desmosome-like zones and digestive vacuoles probably related to digestion of host cell components [30].

Despite the importance of some species of *Hoferellus* as agents of disease in cultured fish, comprehensive data on their development, their molecular diversity and diagnostic tools for the differentiation of pathogenic *vs* non-pathogenic members are inexistent. During our parasitological study of fish and anuran urinary systems, different *Hoferellus* spp. were detected. The main goal of this study was to obtain SSU and ITS rDNA sequences to develop a molecular diagnostic assay differentiating *Hoferellus* spp. but also to determine the phylogenetic relationships between the members of this genus, and clarify the *Hoferellus* species complex infecting cyprinids in the Central European extensive aquaculture. We also provide novel ultrastructural details of plasmodia, evidencing the putatively phagocytic nature of their surface. As a result of the present study, a review of the genus *Hoferellus* is provided, together with its taxonomical emendation in the light of the newly obtained data.

## Methods

### Host sampling sites and collection methods

Cyprinid fish were obtained from eight different localities, ponds and farms in the region of South Bohemia, Czech Republic, between February and November 2011-2013 (Table 1): Common carp *C. carpio* (*n* = 131; total length 2-50 cm, weight 1–2500 g), goldfish *C. auratus* (*n* = 114; total length 2–20 cm, weight 0.6–150 g) and Prussian carp *C. gibelio* (Bloch, 1782) (*n* = 12; total length 10.2–22.5 cm, weight 6–210 g). Peters’ elephantnose fish, *Gnathonemus petersii* (Günther, 1862) (*n* = 10; total length 8–12.5 cm, weight 2.8–9.75 g) imported from Nigeria (Africa), was obtained from a pet shop. All fish were transported alive to the Laboratory of Fish Protistology, at the Institute of Parasitology, euthanized by stunning (60 ppm of clove oil overdose) followed by neural pithing. Urinary system, including kidney, ureters (only in large fishes), and urinary bladder of each fish was dissected. In order to avoid DNA cross contamination, 10 % hydrogen peroxide was used routinely to clean scissors and tweezers during sampling.

**Table 1:** Hosts analyzed within the scope of this study between 2011–2013, comparative prevalence of *Hoferellus* spp. by species and locality as revealed by light microscopy and by single/duplex PCR screening (SSU, ITS) of kidney, ureters and/or urinary bladder and sequence data obtained in this study. All sampled cyprinids originated from the Czech Republic; *Gnathonemus petersii* originated from pet shop (imported from Nigeria); frogs (*Hyperolius* spp.) were sampled separately in 2010 in Kenya

*Hoferellus* species	Host	Location	Coordinates	Prevalence				Sequences length and GenBank Acc. No.	
				Microscopy (Kid, Ure + UB)	PCR (Kid, Ure + UB; Total)			Partial SSU	ITS region
*Hoferellus carassii*	*Carassius auratus* (*n* = 114)	Chřešťovice	49°19’25.32“N; 14°17’10.68”E	14 % (12/86), 33,3 % (23/69)	16.3 % (7/43), 51.5 % (17/33); 51.1 % (23/45)			1969 bp (KU141399)	-
		Bavorov	49° 7’17.58“N; 14° 4’42.13”E	0 % (0/14), 14,3 % (1/7)	7.7 % (1/13), 2/3; 23.1 % (3/13)			-	-
		Jihlava	49°23’56.32“N; 15°33’33.33”E	30 % (3/10), 50 % (3/6)	50 % (4/8), 2/3; 50 % (4/8)			-	799 bp (KU141423)
		Tourov	49°07’22“N; 14°02’04”E	0/4, -	0/4, -; 0/4			-	-
	*Carassius gibelio* (*n* = 12)	Jihlava	49°23’56.32“N; 15°33’33.33”E	0 % (0/12), 58.3 % (7/12)	0/2, 3/3; 3/3			2060 bp (KU141400)	-
					*H. cyprini* PCR (Kid, Ure + UB; Total)	*Hoferellus* sp. PCR (Kid, Ure + UB; Total)	PCR total mixed infections		
*Hoferellus cyprini & Hoferellus* sp.	*Cyprinus carpio* (*n* = 131)	Jindřichův Hradec	49° 9’50.10“N; 15°11’42.20”E	13.8 % (8/58), 22.4 % (11/49)	42.85 % (3/7), 60 % (6/10); 53.3 % (8/15)	7.7 % (1/13), 27.3 % (3/11); 19.1 % (4/21)	25 % (3/12)	782 bp (KU141405)	638 bp (KU141412) 638 bp (KU141413) 648 bp (KU141411) 652 bp (KU141410)
		Chřešťovice	49°19’25.32“N; 14°17’10.68”E	13.5 %(7/52), 2.6 % (1/38)	44.4 % (4/9), 0/7; 26.7 % (4/15)	12.12 % (4/33), 5 % (1/20); 11.8 % (4/34)	13.3 % (2/15)	2046 bp (KU141401)	796 bp (KU141406) 784 bp (KU141421) 785 bp (KU141417) 790 bp (KU141418) 784 bp (KU141422)
		Třeboň	48°59’57.89“N; 14°46’17.14”E	14.3 % (1/7), 14,3 % (1/7)	1/1, 50 % (3/6); 50 % (3/6)	20 % (1/5), 50 % (3/6); 28.6 % (2/7)	16.6 % (1/6)	796 bp (KU141403)	625 bp (KU141414)
		Nakolice, Nové Hrady	48°47’38.68“N; 14°49’53.82”E	20 % (1/5), 20 % (1/5)	0/2, 60 % (3/5); 60 % (3/5)	0/5, 0/5; 20 % (1/5)	20 % (1/5)	854 bp (KU141404)	605 bp (KU141419) 606 bp (KU141420) 796 bp (KU141407)
		Fish market (České Budějovice)		0/4, 0/4	0/2, -; 0/2	2/4, -; 2/4	0/2	-	-
		Bavorov	49° 7’17.58“N; 14° 4’42.13”E	1/3, 0/1	1/1, -; 1/2	0/2, -; 0/2	0/2	-	-
		Jihlava	49°23’56.32“N; 15°33’33.33”E	0/2, 1/2	-, 1/1; 1/2	-, 1/1; 1/2	1/2	1995 bp (KU141402)	798 bp (KU141408) 798 bp (KU141416) 625 bp (KU141409) 797 bp (KU141415)
*Hoferellu*s *gnathonemi* sp. n.	*Gnathonemus petersii* (*n* = 10)	Petshop	NA	10 % (1/10) (kidney)	10 % (1/10) (kidney)			2162 bp (KU141398)	.
*Hoferellus anurae*	*Hyperolius kivuensis* (*n* = 7)	Kakamega, Kenya	0°20’56“N, 34°51’55.9”E	57 % (4/7)	-			964 bp (KU141397)	-
	*Hyperolius viridiflavus* (*n* = 10)	Kakamega, Kenya	0°20’56“N, 34°51’55.9”E	40 % (4/10)	-			-	-

A total of 17 adult male reed frogs of the genus *Hyperolius* (Hyperoliidae) were examined: *Hyperolius kivuensis* Ahl, 1931 (*n* = 7), *Hyperolius viridiflavus* (Duméril et Bibron, 1841) (*n* = 10). All frogs were collected in November 2010 in/around a small roadside pond ~420 m south-east of Udo’s campsite, Kakamega forest reserve, Kenya; 0°20’56“N, 34°51’55.9”E, 1600 m a.s.l. Frogs were euthanized by pithing and examined for myxosporeans within 24 h after collection. Prior to dissection, dorsal-, ventral- and lateral-view photographs of live and/or freshly dead, individual frogs were taken for identification purposes. Samples (1–2 mm each) of kidneys were dissected and preserved in 10 % buffered formalin and absolute ethanol, stored for several months and processed routinely for histology and DNA sequencing, respectively. Vouchers of processed frogs were deposited in the herpetological section of the National Museums of Kenya in Nairobi under accession numbers A5264/1–7 (*H. kivuensis*), A5265/1–10 (*H. viridiflavus*).

All animal procedures were performed in accordance with Czech legislation (section 29 of Act No.246/1992 Coll. on protection of animals against cruelty, as amended by Act No. 77/2004 Coll.). We declare that animal handling complied with the relevant European and international guidelines on animal welfare, namely Directive 2010/63/EU on the protection of animals used for scientific purposes and the guidelines and recommendations of the Federation of Laboratory Animal Science Associations. Permit NCST/PRI/12/1/BS/204 for collection of amphibian samples was issued by The National Council for Science and Technology, Nairobi, Kenya; institutional affiliation was granted by National Museums of Kenya (NMK/ZLG/TRN/6/1.2); field work in nature and forest reserves was approved by Kenya Wildlife Service (KWS/5001) and Kenya Forest Service (RESEA/1/KFS (6)), respectively.

### Morphological analysis

Fresh smears of urinary bladder, ureters and kidney were examined using light microscopy at 400x magnification to detect the presence of plasmodia and/or spores of *Hoferellus* spp. Digital images of fresh spores and plasmodia were taken at 1000x magnification. Measurements of 30 spores of *H. carassii*, 31 spores or *H. cyprini*, 10 spores of *Hoferellus gnathonemi* sp. n. in *G. petersii* and 50 spores of *H. anurae* were taken from digital images using the computer software ImageJ 1.47v (National Institutes of Health, USA) and calibrated against a digital image of a graticule. Morphological measurements of spores followed the recommendations of [25, 31]: spore length and width, spore posterior width, polar capsule length and width, and caudal filaments length. Plasmodia length and width were measured from 15 plasmodia of *H. carassii*, 36 plasmodia of *H. cyprini*, 10 plasmodia of *Hoferellus gnathonemi* sp. n. in *G. petersii* and 10 plasmodia of *H. anurae*. All measurements are given in μm as means ± standard deviation with range in parentheses. Spores measured using light microscopy as well as organ samples with or without microscopically detectable plasmodia were used for DNA extraction and sequencing of different regions of rDNA. One replicate of *H. carassii* spores found in *C. auratus* was used for scanning electron microscopy. For histology, 10 % formalin preserved samples of cyprinid fishes and frog kidneys were processed routinely, embedded in paraffin, sections stained with hematoxylin and eosin (H&E) and examined as specified above.

### Electron microscopy

For scanning electron microscopy (SEM), *H. carassii* spores from an infected *C. auratus* urinary bladder smear were washed with 0.1 M sodium cacodylate buffer (pH 7.2), collected and fixed with 2.5 % glutaraldehyde in cacodylate buffer. The day of processing, the parasites were washed in cacodylate buffer and centrifuged (800 *g*, 5 min). Thereafter, the parasites were left to settle onto an ethanol-washed and 0.1 % poly-D-lysine coated coverslips for 30 min and then fixed for 30 min using 2.5 % glutaraldehyde in cacodylate buffer. After rinsing in the same buffer (15 min) the parasites on the coverslip were post-fixed with 1 % osmium tetroxide in 0.1 M sodium cacodylate buffer for 30 min. Coverslips were then washed for 15 min in distilled water, dehydrated in an ascending alcohol series and critical-point dried. Thereafter, the coverslips were mounted on stubs, gold sputtered-coated and examined with a JEOL JSM-7401 F (JEOL Ltd., Japan).

For transmission electron microscopy (TEM), selected heavily parasitized paraffin embedded pieces of frog kidneys used previously for histology were first deparaffinized as follows: incubation of the whole paraffin block in 37 °C till complete melting of the paraffin, xylene bath – twice for 1 h, 96 % ethanol bath – twice for 1 h, 70 % ethanol bath – twice for 1 h. Tissue samples devoid of paraffin were then post-fixed in freshly prepared 2.5 % glutaraldehyde in 0.1 M phosphate buffer. The specimens were then washed for 1 h in the same buffer, post-fixed in 1 % osmium tetroxide in the same buffer for 3 h and dehydrated in an alcohol series, before embedding in Epon resin (Polybed 812). Sections were cut with diamond knives and stained with uranyl acetate and lead citrate. Observations and imaging were performed using a JEOL 1010 TEM.

### rDNA sequencing and cloning

Samples of kidney, ureters and urinary bladders with spores and/or plasmodia of two goldfish, one Prussian carp, five common carp, one Peters’ elephantnose fish and one reed frog, *H. kivuensis* (see Table 1) were stored in 400 μL of TNES (10 mM Tris-HCl (pH 8), 125 mM NaCl, 10 mM EDTA, 0.5 % SDS, 4 M urea) [32]. DNA was digested with 100 μg/ml of proteinase K, overnight at 55 °C, and extracted following a simplified phenol-chloroform protocol [1]. The extracted DNA was re-suspended in 100 μL of RNAase/DNAase free water and left to dissolve overnight at 4 °C.

Partial SSU rDNA sequences and ITS regions (ITS1, 5.8S and complete ITS2 sequence) were amplified using different primer combinations and PCR conditions (Table 2) [2, 33–36]. SSU rDNA amplicons were obtained using primers Erib1 + Erib10. If this PCR failed or abundant host tissue was present, a nested PCR with MyxospecF + MyxospecR or MyxGP2F + Act1R was performed. The ITS region was amplified using HofSSUend + ITS-Zschok-Rev. PCRs were conducted in 25 μl reactions with 0.025Uμl^−1^ Titanium Taq DNA polymerase and 10× buffer which contained 1.5 mM MgCl_2_ (BD Biosciences Clontech), with 0.2 mM of each dNTP, 0.5 mM of each primer, and 10–150 ng of template DNA. The PCR cycle conditions consisted of denaturation 95 °C 3–5 min, followed by 30–40 cycles of amplification: 94 °C for 1 min, specific annealing temperature (Table 2) for 1–1 min 30 s, and 68 °C for 1–2 min, and final extension at 68 °C for 8–10 min. After checking for the presence of the expected DNA amplicons in a 1 % agarose gel in sodium acetate buffer, PCR products were purified for sequencing using a Gel/PCR DNA Fragments Extraction Kit (Geneaid Biotech Ltd., USA). Preferably, direct sequencing of PCR products was attempted. Problematic amplicons were cloned into the pDrive Cloning vector (Qiagen PCR Cloning Kit, Germany) and transformed into the competent *E. coli* strain XL-1. Plasmid DNA was isolated using a High Pure Plasmid Isolation Kit (Roche Applied Science, Germany). PCR products or plasmids were sequenced on an ABI PRISM 3130x1 automatic sequencer (Applied Biosystems, Czech Republic). The overlapping partial corresponding sequences of SSU rDNA and ITS regions were assembled into single contigs in SeqMan II v5.05 (DNASTAR Inc., Madison, Wisconsin).

**Table 2:** Name of primers, ribosomal gene target region, primers’ sequence, amplicon size base pairs, PCR conditions, primers used for sequencing and reference

Primer name	Target rDNA	Sequence 5’-3’	Amplicon size (bp)	Annealing temperature (°C)	PCR round	Sequencing	Author
Erib1	SSU	ACCTGGTTGATCCTGCCAG	≈1900	60	1^st^	Yes	[33]
Erib10	SSU	CTTCCGCAGGTTCACCTACGG				Yes	[33]
MyxospecF	SSU	TTCTGCCCTATCAACTWGTTG	≈900	52	Nested	Yes	[2]
MyxospecR	SSU	GGTTTCNCDGRGGGMCCAAC				Yes	[2]
MyxGP2F	SSU	WTGGATAACCGTGGGAAA	≈800	58	Nested	Yes	[34]
Act1R	SSU	AATTTCACCTCTCGCTGCCA				Yes	[35]
HofcarasF	SSU	GTGTTCTCACGAATGTGTAT	≈300	54	Duplex	No	Present study
HofcarasR	SSU	AACCTATAAGGCTATTATCTG				No	Present study
HofK41F	SSU	TTGTGTATATTATGTAATGTATTG	≈900			No	Present study
HofK41R	SSU	CATCTTGTTACCAAAATAAC				No	Present study
HofK48F	SSU	ACGTATGTGTGTTATAATGTGTATG	≈1400	56	Single	No	Present study
HofK48R	SSU	TTTGTTGCCAAAACAACCAC				No	Present study
HofSSUend	SSU	GTGTACTTCATAAAAGTACGC	≈700	60	Single	Yes	Present study
ITS-Zschok-Rev	LSU	GATTCTCATAGTAACTGCGAGTG				Yes	[36]
HofK102R	ITS	GCACCACAAAAACATTACTT	≈200 (with HofSSUend)	55	Single	No	Present study
HofK107R	ITS	CATGCACCACACAAATTAT	≈200 (with HofSSUend)	55	Single	No	Present study

### Specific primer design for PCR-based diagnostics of *Hoferellus* spp

Based on the *Hoferellus* SSU rDNA sequences obtained from *C. auratus* and *C. carpio* and their alignment with other myxozoan species belonging to the same phylogenetic clade (“freshwater clade” [2]), specific primers (HofcarasF/R and HofK41F/R; Table 2) were designed for a duplex PCR assay. Another PCR assay was designed for the *Hoferellus* sp. detected in common carp (HofK48F/R). Based on the different *Hoferellus* ITS region genotypes obtained in *C. carpio* specific reverse primers (HofK102R and HofK107R, Table 2) were used, combined with the forward primer HofSSUend, in a single-round duplex PCR assay. All specific primers were designed using NCBI\Primer-Blast (National Center for Biotechnology Information, [37]). No cross-reaction was detected for the new primers designed. Primers were tested for their optimal annealing temperature in a gradient PCR and subsequently applied in its specific PCR assay. Specific PCR assays were conducted as before but in 10 μl reactions with 0.4 U Taq-Purple DNA polymerase and 10× buffer, which contained 1.5 mM MgCl_2_ (Top-Bio, Czech Republic) and 72 °C for extension. For diagnosis of other *Hoferellus* genotypes, the same PCR protocol was used but with Titanium Taq DNA polymerase, at 68 °C extension temperature. Thereafter, all PCR products were submitted to electrophoresis and positive/negative samples were recorded.

### Phylogenetic analyses

Two main alignments were created using MAFFT v6.864 [38] with a L-INS-i strategy and default parameters. Alignments contained newly obtained and published myxozoan sequences retrieved from GenBank. The SSU rDNA alignment contained 2880 characters, whereas ITS region alignment contained 1168 characters. Phylogenetic analyses were performed using maximum likelihood (ML), maximum parsimony (MP) and Bayesian inference (BI). ML was done in RAxML v7.0.3. [39] with the GTR GAMMA model of evolution. MP was performed in PAUP*v4.0b10 [40] with heuristic search with random taxa addition and the TBR swapping algorithm. All characters were treated as unordered, Ts:Tv ratio was set to 1:2 and gaps were treated as missing data. BI was computed in the MrBayes v3.0 [41] with the GTR + Γ + I model of evolution. Posterior probabilities were calculated over 1,000,000 generations via two independent runs of four simultaneous Markov chain Monte Carlo with every 100th tree saved. Tracer v1.4.1 [42] was used to ascertain the length of burn-in period. For ML and MP, the bootstrap supports were calculated from 500 replicates. Genetic distances (in %) were computed in PAUP* v4.0b10 with default P parameter from the SSU rDNA and ITS region alignments. Both alignments were adjusted: 5’ and 3’ ends were cut in order to have sequences of the same length and the inserts in *Myxidium streisingeri* Whipps et al., 2014 (Acc. Num. KM001684) were excluded from the alignment, which included 864 and 860 characters, respectively.

## Results

### Prevalence of *Hoferellus* spp

Plasmodia of *Hoferellus* spp. in the lumina of the kidney tubules were observed with a prevalence of 13.2 % (15/114) in *C. auratus* from two localities and 13.8 % (18/131) in *C. carpio* from five localities, but were not observed in kidney tubules of *C. gibelio* (0/12). The plasmodia in the kidney tubules were usually smaller than those in ureters and urinary bladder, possessing refractile granules and occasionally forming spores. Larger pre-sporogonic and sporogonic stages, as well as free mature spores were observed in ureters and urinary bladder with a prevalence of 33 % (27/82) in *C. auratus* from three localities, 14.2 % (15/106) in *C. carpio* from five localities and 58.3 % (7/12) in *C. gibelio* from Jihlava (Table 1). These stages were sometimes motile, with large hyaline areas and pseudopodia. In general, microscopic infection prevalence in all goldfish was higher in the ureters/urinary bladders than in kidneys. In common carp, the infection prevalence was similar in kidneys and ureters/urinary bladders, whereas in Prussian carp, only urinary bladders were infected (Table 1).

In common carp, mature spores were only found in 2.3 % (3/131) of the fish examined. The earliest spores were detected in the urinary bladder, in February (Třeboň, 1 fish) and in the kidney and urinary bladder in April-May (Chřešťovice and Jindřichův Hradec, 1 fish each). In goldfish, mature spores were detected with a prevalence of 10.5 % (12/114, Chřešťovice and Jihlava): 7 kidneys in March-April and June, and 5 urinary bladders in June-August. No obvious signs of disease or kidney enlargement or intracellular stages were observed in any cyprinid fish at any time.

Spores matching the characteristics of *Hoferellus* located within large plasmodia were observed in the kidney tubules with a prevalence of 10 % (1/10) in *G. petersii*. The morphology of these spores did not match that of other *Hoferellus* spp. and hence is described as a new species below.

In wet mounts of kidneys from reed frogs, spores matching original description of *Hoferellus anurae* Mutschmann, 2004 described from *Afrixalus* and *Hyperolius* spp., were observed with a prevalence of 40 % (4/10) and 57 % (4/7) in *H. viridiflavus* and *H. kivuensis*, respectively. Fresh mounts and histology revealed the same prevalence, but plasmodia were only discernible in histological sections.

### Morphology of *Hoferellus* spp. from cyprinids

After comparison with previous reports and based on morphological and morphometrical data (see Tables 3 and 4), we ascribed the spores found in *C. auratus* to *H. carassii* and the spores found in *C. carpio* to *H. cyprini*.

**Table 3:** Reference, hosts, geographic location, prevalence, spore and plasmodia measurements (in μm) from all reports to date on *Hoferellus carassii* Akhmerov, 1960

*Hoferellus carassii* Akhmerov, 1960	*Mitraspora cyprini* (Fujita, 1912)	*Hoferellus carassii* Akhmerov, 1960	*Mitraspora cyprini* (Fujita, 1912)	*Mitraspora cyprini* (Fujita, 1912)	*Sphaerospora cyprini* (Fujita, 1912)	*H. carassii* Akhmerov, 1960	*H. carassii* Akhmerov, 1960	*H. carassii* Akhmerov, 1960	*H. carassii* Akhmerov, 1960	*H. carassii* Akhmerov, 1960
Reference	[9]	[22]	[23]	[17]	[24]	[12]	[12]	[25]	[26]	Present study
Host/s	*C. auratus C. carpio*	*C. gibelio*	*C. carassius C. auratus*	*C. auratus*	*C. carassius*	*C. auratus*	*C. gibelio*	*C. auratus*	*C. gibelio*	*C. auratus*
Geographic location	Japan	Russia	Japan	Japan	Spain	USA	Slovakia	Germany	China, Russia	Czech Republic
Prevalence	-	-	-	-	3.7 %	-	11.8 % (2/17)	-	-	23.1-51.1 %
Host location	Renal tubules of kidney and ureters	Renal tubules	Occasionally intracellular	Intracellular and intraluminal renal tubules	Urinary bladder	-	Renal tubules	Urinary bladder and ureter	Kidney and renal tubules	Kidney, ureters and urinary bladder
Spore shape	Monk’s hood, slightly attenuated at the posterior end	Goblet-like	-	Monk’s hood, spindle-shaped in profile; ovoid from above	Oval	Miter like, elongated, rarely almost oviform	Appear less elongated than in *C. auratus auratus*	Long and almost oviform	Sharp anterior end, round posterior end	Miter-like, sometimes round
Spore length	10-13	12	11.5-16.9	12.2 (11.2-14)	11-14	13 (11-15.2)	10.6 (9.5-11.4)	13.07 ± 1.06	12	13.1 ± 2.1 (10.2-17.3)
Spore width	5	6-6.5	6.3-10.2	6.3 (5.6-7)	9-10	7.5 (6.2-9)	6 (5-6.6)	8.44 ± 0.64	6-6.5	9.6 ± 1.2 (7.6-11.8)
Thickness	-	6-6.5	4.3-7.1	6.1 (4.3-7)	7-8	4.6 (4.2-6)	3.8 (3.6-4.5)	7.58 ± 0.67	6-6.5	7.1-7.4
Length of polar capsules	3.8	5.3-5.5	3.2-4.9	4 (2.4-4.2)	3.5-5	4.2 (3.3-5)	4.8 (4.4-5)	3.89 ± 0.34	5.3-5.5	4 ± 0.7 (2.8-5.8)
Width of polar capsules	2	2.6-2.8	1.7-2.3	2.9 (2.6-2.9)	2-4	2.4 (2-2.6)	2.8 (2.6-3)	2.33 ± 0.2	2.6-2.8	2.4 ± 0.5 (1.8-3.6)
Length of polar filaments-	15	-	-	53.8 (35-77)	35	-	-	-	-	-
Coils of polar filament	-		-	-	7-8	5-6	5 (4-6)	-	-	6
Posterior width	-		-	-	-	-	-	4.12 ± 0.4	-	6.3 ± 0.7 (5.2-7.9)
Length of caudal filaments	5.8		4.2-11.1	5.8 (4.2-7)	4.5-7	4.5-6	-	4.01 ± 0.56	-	5.4 ± 1.2 (3.9-8)
Number of caudal filaments /ridges	8/8	20/-	8/8	8 (7-10)/8(7-10)	7-8/6	18-22	-/18-20	-	20/8-10	10/14-15 per valve
Plasmodia length	-	-	12-169	27.5 (16-129)	38-42.5	-	40	18.56 ± 4.3	-	27.9 ± 8.8 (13.5-41)
Plasmodia width	-	-	-	24.6 (13.6-61.2)	15.5-17	-	22	12.21 ± 2.39	-	19.9 ± 5.5 (12.5-30.2)
Spores per plasmodia	3-4	-	1-4	6 (3-21)	3-6	-	Up to 4	-	-	1-3

**Table 4:** Reference, hosts, geographic location, prevalence, spore and plasmodia measurements (in μm) from all reports to date on *Hoferellus cyprini* (Doflein, 1898)

*Hoferellus cyprini* (Doflein, 1898)	*Hoferia cyprini* (Doflein, 1898)	*Hoferellus cyprini* (Doflein, 1898)	*Mitraspora cyprini* (Fujita, 1912)	*Mitraspora cyprini* (Fujita, 1912)	*Hoferellus cyprini* (Doflein, 1898)	*Mitraspora cyprini* (Fujita, 1912)	*Hoferellus cyprini* (Doflein, 1898) Berg, 1898	*Hoferellus cyprini* (Doflein, 1898)	*Hoferellus cyprini* (Doflein, 1898)
Reference	[43]	[44]	[9]	[45]	[45]	[46]	[12]	[13]	Present study
Host/s	*C. carpio*	*C. carpio*	*C. carpio*	*C. carpio*	*C. carpio*	*C. carpio*	*C. carpio*	*C. carpio*	*C. carpio*
			*C. auratus*						
Geographic location	Germany	France	Japan	Japan	France, Germany	Germany	Czechoslovakia	Hungary	Czech Republic
Prevalence	-	-	-	-	-	-	Up to 32 %	25 % (1/4)-66.6 % (2/3)	26.7-60 %
Host location	Renal tubules	Renal tubules	Renal tubules kidney, ureters	Renal tubules kidney, ureters	Young trophozoites in epithelium, adults in urinary tubules	Renal tubules, ureters	Renal tubules	Renal tubules, intraepithelial stages	Renal tubules kidney, ureters, urinary bladder
Spore shape	Pyramidal	Truncated pyramid	Monk’s hood, slightly attenuated at the posterior end	As Fujita 1912, more rounded	Pyramidal with 2 short tail like processes at post end	Anterior pointed, posterior truncated	Bullet-like, stubby	Short ellipsoidal (stubbier)	Short bullet-like, slightly round
Spore length	10-12 (incl. tails)	10-12	10-13	10	10-12	9.5-12	9 (8-10.2)	9 (8.5-10)	8.5 ± 0.8 (7.4-10.4)
Spore width	8	6-8	5	8-9	8	6.5-7.5	6.5 (6-7)	6.6 (5.2-7.1)	6.7 ± 0.6 (5.2-7.7)
Thickness	-	-	-	6-8	-	-	Similar to width	5.6 (5.2-5.8)	4.8-5.5
Length of polar capsules	3	3	3.8	4	3	4-5	3.6 (3.3-4)	3.8 (3.5-4.2)	3.1 ± 0.4 (2.1-3.9)
Width of polar capsules	-	-	2	1.5-2	-	2-2.5	2.4 (2.3-2.5)	2.15 (2.1-2.2)	2.1 ± 0.3 (1.6-2.9)
Length of polar filaments-	-	-	15	35-40	-	-	-	-	31.3 (29.5-32.4)
Coils of polar filament	-	-	-	-	-	4-5	5	4-5	5 (4-6)
Posterior width	-	-	-	-	-	-	-	-	4.2 ± 0.9 (2.4-5.7)
Length of caudal filaments	2	1.5-2	5.8	5-6	2	3-5	2-4	4.5-5	4.1 ± 0.7 (3.4-6.2)
Number of caudal filaments /ridges	-/9-10	-	8/8	5-6/ variable	-/9-10	7-9/7-9	-/20-24	20/20	10/- per valve
Plasmodia length	20-30	20-30	-	10-40	20-30	15-40 (70)	65	6-8 (young) intracelular	33.2 ± 11.9 (12.5-70.8)
								15-40 (in lumen)	
Plasmodia width	-	-	-	-	-	8-27	40	15-30 (in lumen)	17.8 ± 5 (10.1-30.4)
Spores per plasmodia	2	-	3-4	2	Polyspororous	1-9	Several to 10-12	3-10	1-6 spores

*Hoferellus carassii* Akhmerov, 1960.

#### Morphology of spores

**Fig. 1:**
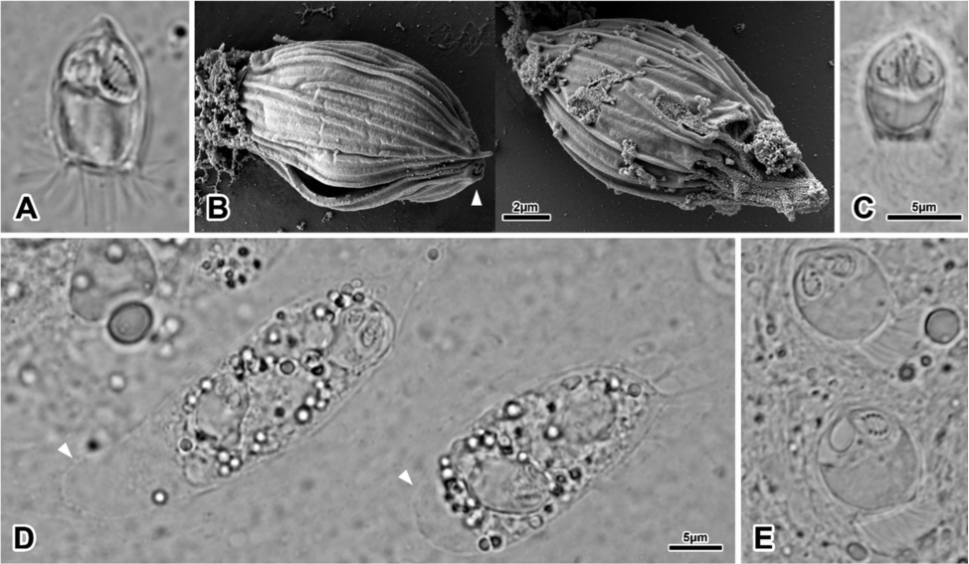
*Hoferellus* spp. spores and plasmodia in cyprinid fishes (LM & SEM). **a** *Hoferellus carassii* mature spores from the urinary bladder of *C. auratus*; **b** Polar capsule openings (arrowhead) at the anterior end of the *H. carassii* spore and longitudinal ridges pattern on the valve surface; **c** *Hoferellus cyprini* mature spores from the urinary bladder of *Cyprinus carpio*; **d** Two amoeboid plasmodia of *H. cyprini* in the urinary bladder of *C. carpio*, showing large hyaline areas (arrowheads); **e** *H. carassii* immature spores from the urinary bladder of *C. auratus*

**Fig. 2:**
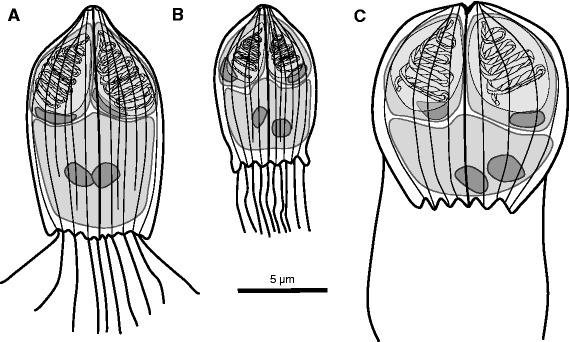
Composite line drawings of **a** *Hoferellus carassii*, **b** *Hoferellus cyprini* and **c** *Hoferellus gnathonemi* sp. n

Miter-like spores, elongated with pointed anterior end and truncated posterior end (Fig. 1a, 2a), 13.1 (10.2–17.3) in length, 9.6 (7.6–11.8) in width, 7.1–7.4 in thickness and 6.3 (5.2–7.9) in posterior width. Two valves joined by longitudinal suture, each possessing at least 14–15 longitudinal ridges, which are sometimes bifurcated and/or incomplete (Fig. 1b). Only 10 per valve continued into caudal filaments. Irregular posterior edge corresponding to ridges extending into caudal filaments. Posterior part of spore possessing two distinct but relatively small projections, one on each valve. Single, binucleate sporoplasm. Polar capsules pyriform, 4 (2.8–5.8) in length and 2.4 (1.8–3.6) in width. Polar filament allocated in 6 coils, usually oblique to the longitudinal axis of capsule (Fig. 1a). Polar capsule openings at anterior end, on top of conical protuberances of each valve. (Fig. 1b).

#### Localization and morphology of plasmodia

Amoeboid round and pyriform plasmodia in lumina of kidney tubules, ureters and urinary bladder, 27.9 (13.5–41) in length and 19.9 (12.5–30.2) in width, with relatively large hyaline area, 5.7 (3.6–7.9) in width. Polysporic plasmodia, forming up to 3 spores. Spores within plasmodia never observed in pairs.

*Hoferellus cyprini* (Doflein, 1898).

#### Morphology of spores

Short bullet-like spores, slightly round and stubby, with pointed anterior end and truncated posterior end (Fig. 1c, 2b), 8.5 (7.4–10.4) in length, 6.7 (5.2–7.7) in width, 4.8–5.5 thickness and 4.2 (2.4–5.7) in posterior width. Two valves, longitudinal suture. Ten caudal filaments per valve. Serrate posterior end corresponding to ridges extending into caudal filaments. Posterior part of spore possessing two distinct but relatively small projections, one on each valve. Binucleate, single-cell sporoplasm. Polar capsules pyriform, 3.1 (2.1–3.9) in length and 2.1 (1.6–2.9) in width. Polar filament in 4–6 coils, longitudinally oriented to the capsule (Fig. 1c).

#### Localization and morphology of plasmodia

Amoeboid, round and pyriform plasmodia in lumina of kidney tubules, ureters and urinary bladder (Fig. 1d), 33.2 (12.5–70.8) in length and 17.8 (10.1–30.4) in width, with hyaline area, 7.3 (2.3–20.2) in width. Polysporic plasmodia, forming up to 6 spores. Spores within plasmodia never observed in pairs.

#### Remarks

Anterior pole of spore end in *H. cyprini* less pointed than in *H. carassii*. The irregular posterior spore end, as well as the two posterior small valvular projections are more prominent in *H. cyprini* than in *H. carassii*. In both species, spores in different degrees of maturation were observed. Mature spores were more compact and elongate (less round), with clear sporoplasm, fully formed polar capsules and visibly coiled polar filament. Immature spores (Fig. 1e) were more round, less compact and polar capsules were not always in their definitive position and/or the coiled polar filament was not visible.

#### *Hoferellus* sp. ex *C. carpio*

In common carp, a second hoferellid species was detected using molecular tools only (see section “rDNA sequences and phylogenetic results” below) in mixed infection with *H. cyprini*. No morphology could be ascribed to this genotype.

### Description of *Hoferellus gnathonemi* sp. n

Phylum Cnidaria Hatschek, 1888

Unranked subphylum Myxozoa Grassé, 1970

Class Myxosporea Bütschli, 1881

Order Bivalvulida Schulman, 1959

Suborder Variisporina Lom et Noble, 1984

Family Myxobilatidae

Genus *Hoferellus* Berg, 1898

*Hoferellus gnathonemi* sp. n.

***Morphology of spores***

**Fig. 3:**
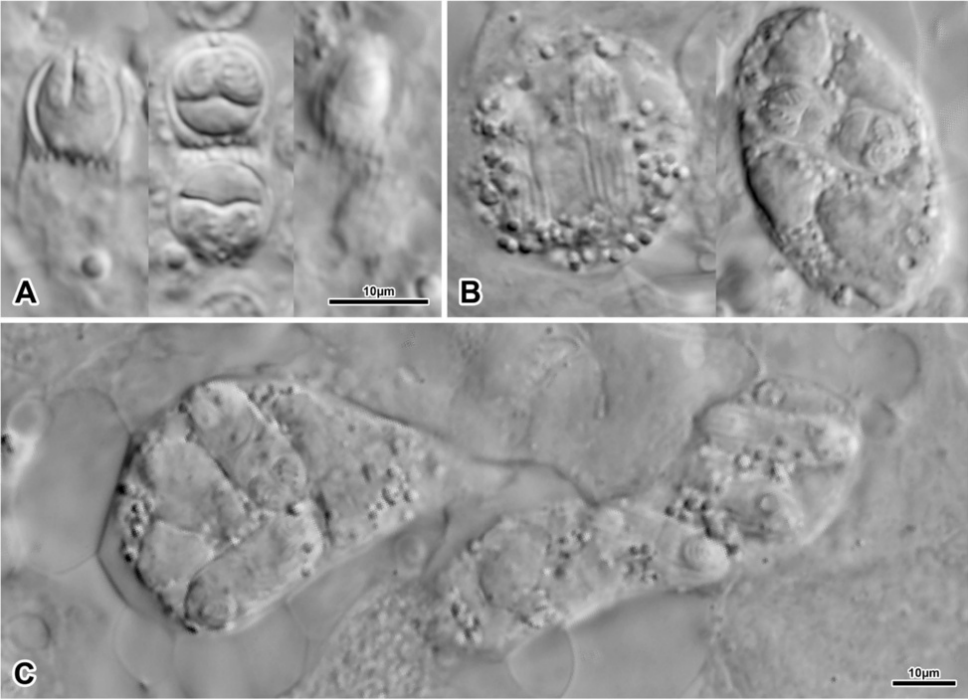
*Hoferellus gnathonemi* sp. n. from *Gnathonemus petersii* (LM). **a** Wet mount of kidney showing single spore with characteristic posterior projections and their typical arrangement in disporous plasmodia, and with spores showing distinct longitudinal ridges; **b** Individual spherical and subspherical plasmodia; **c** A group of plasmodia, containing disporous sporoblasts, inside the renal tubule. B and C in the same scale

Round myxospores (Fig. 2c, 3a), 11.9 (10.3–14.3) in length, 11 (9.9–12.7) in width and 8.1 (6.3–9.4) in posterior width. Two valves, joined by longitudinal suture, 6–8 longitudinal ridges visible on spore surface in lateral view. Two distinct posterior caudal filaments, one on each valve, 7.2–8.2 in length. Caudal filaments considered absent. Posterior end of spore valves serrated. Single, binucleate sporoplasm. Polar capsules pyriform to ovoid, 5.8 (3.7–7.9) in length and 3.7 (2.7–4.8) in width. Polar filament allocated in 3 to 4 coils. Spores developing typically inside disporous sporoblasts with spores facing their posterior ends. Once released from plasmodia, some spores remain attached to each other.

#### Localization and morphology of plasmodia

Amoeboid round and pyriform plasmodia in kidney tubules (Fig. 3b-c), 55.6 (42.6–89.3), in length and 37 (23.5–44.4) in width, containing abundant refractile granules. Plasmodia polysporic, with disporous sporoblasts and up to 6 spores per plasmodium. Spores within plasmodia observed in pairs.

### Taxonomic summary

Type host: Peter’s elephantnose, *Gnathonemus petersii* (Günther, 1862) (Osteoglossiformes: Mormyridae).

Type locality: Nigeria, Africa (not exact location, fish obtained from pet shop).

Site: kidney tubules.

Prevalence: 10 % (1/10) *G. petersii*.

Etymology: The species name derived from generic name of type host.

Material deposited: DNA sample deposited at the protistological collection of the Institute of Parasitology, Biology Centre, Czech Academy of Sciences, České Budějovice, no. IPCAS ProtColl 33.

Sequence data: 2162 bp of SSU rDNA sequence obtained from one infected *G. petersii* (GenBank Acc. Num. KU141398)

#### Remarks

Single free spores showed two clear posterior filaments (Fig. 3a). The serrated posterior ends of the spores suggest that caudal filaments might be present, generally indiscernible in light microscopy but potentially indicated in Fig. 3a (right spore). Further material, preferably processed for SEM, is needed to confirm the presence/absence of caudal filaments in this new species.

### Redescription of *Hoferellus anurae* Mutschmann, 2004 

#### Morphology of spores

**Fig. 4:**
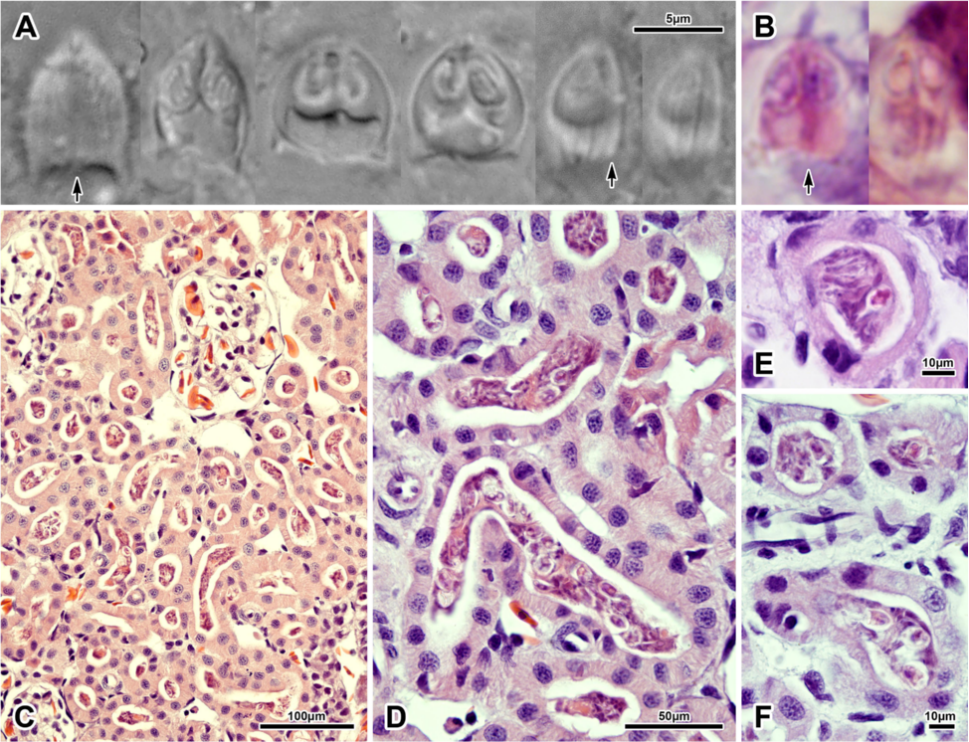
*Hoferellus anurae* from *Hyperolius* spp. from Kenya (LM). **a**-**b** Spore morphology and shape variability, distinct sutural (arrows) and longitudinal valvular ridges as seen in fresh mounts (**a**) and histological sections (**b**); **c** Paraffin section showing restriction of plasmodia to renal tubules and their absence in glomerular spaces, **d** longitudinal and **e**, **f** transversal sections of plasmodia partly attached to microvillar zone of tubular epithelium. A and B in the same scale

Spores 8 (7.5–8.5) × 7 (6.0–7.5), miter-shaped with pointed anterior pole, longitudinal ridges and usually discernible longitudinal suture. Posterior part of spore possessing two distinct but relatively small projections 0.5–1.2 long, one on each valve, giving the spore somewhat triangular appearance (Fig. 4a). In a negligible number of spores, much smaller projections corresponding with the end of the longitudinal valvular ridges, not clearly discernible posterior filaments, seemed to be present between the two main posterior projections (Fig. 4a). Posterior filaments otherwise indiscernible by light microscopy and considered absent. Some spores more spherical than others (Fig. 4a), possible reflecting degree of maturation. Single binucleate sporoplasm, or two uninucleate – not discernible in light microscopy (nor in TEM preparations). Polar capsules 4.0 × 2.5, pyriform, filling approximately 1/2 of spore (Fig. 4b), coils of polar filament usually oblique to the longitudinal axis of capsule (Fig. 4a).

#### Localization and morphology of plasmodia

Present only in renal tubules, never observed in glomerular spaces (Fig. 4c). Spore-producing plasmodia very elongate as revealed in longitudinal tubule sections, polysporic – up to 19 spores per section plane observed (Fig. 4d). Firmly attached by relatively small part of their surface to the host tubular epithelium (Figs 4e-f, see below for ultrastructural details). Spores within plasmodia never observed in pairs. No gross pathological changes observed in any sample upon dissection, no obvious pathology observed in histological sections.

#### Intracellular presporogonic stages putatively assigned to H. anurae

**Fig. 5:**
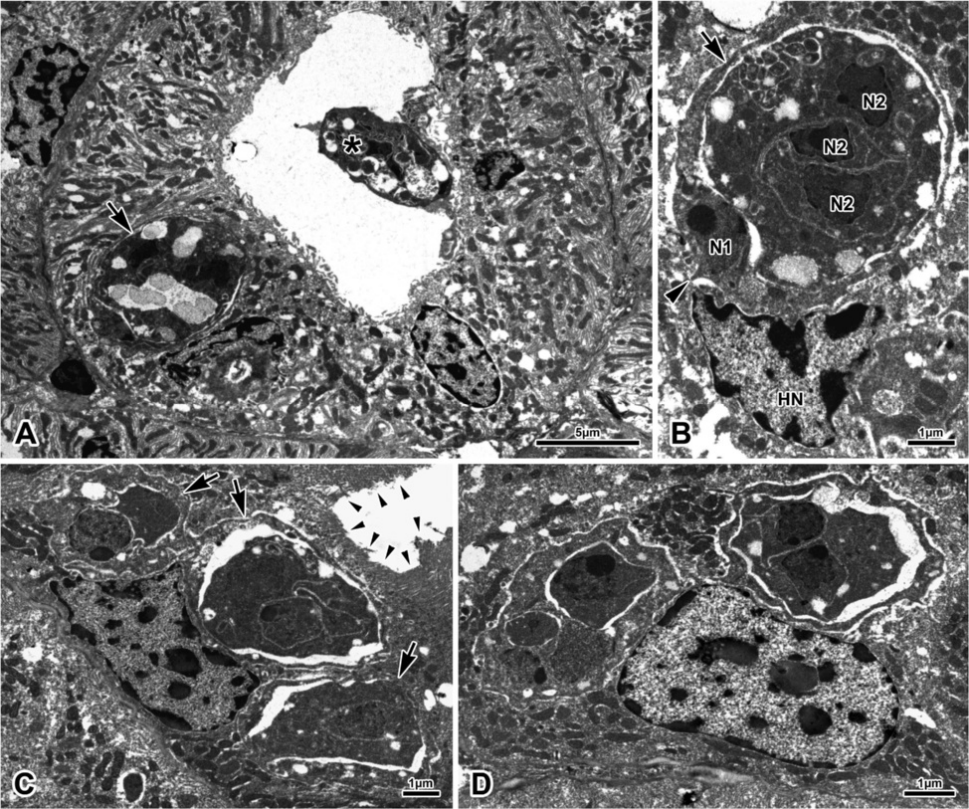
Ultrastructure of intracelullar stages of *Hoferellus anurae* (TEM) (**a**) Intracellular stages (arrows) located in the tubular epithelium, **b** typical cell composition, *i.e.* enveloping primary cell with protoplasmic projections (arrowhead) containing three secondary cells, **c**, **d** overall appearance of intracellular stages; note the unaffected healthy microvillar zone (small arrowheads) of a tubule infected solely with intracellular stages in C. Asterisk = sporogonic plasmodium in tubular lumen, HN = host cell nucleus

Observed regularly in all TEM preparations within epithelial cells of renal tubules, sometimes occupying epithelial cells of tubules containing sporogonic plasmodia in their lumina (Fig. 5a). Although exact cell configuration of these putatively presporogonic stages remained somewhat unclear as serial sections were not analysed, these stages apparently consisted of a single enveloping primary cell, containing several secondary cells (Figs 5b-d). Epithelial cells of tubules containing only these intracelular stages, but devoid of sporogonic histozoic plasmodia, retained a healthy microvillar layer (Fig. 5c). The intracelular stages were never recognized with certainty in corresponding paraffin sections from which the TEM preparations were made.

#### Ultrastructure of coelozoic spore-producing plasmodia and myxospores

**Fig. 6:**
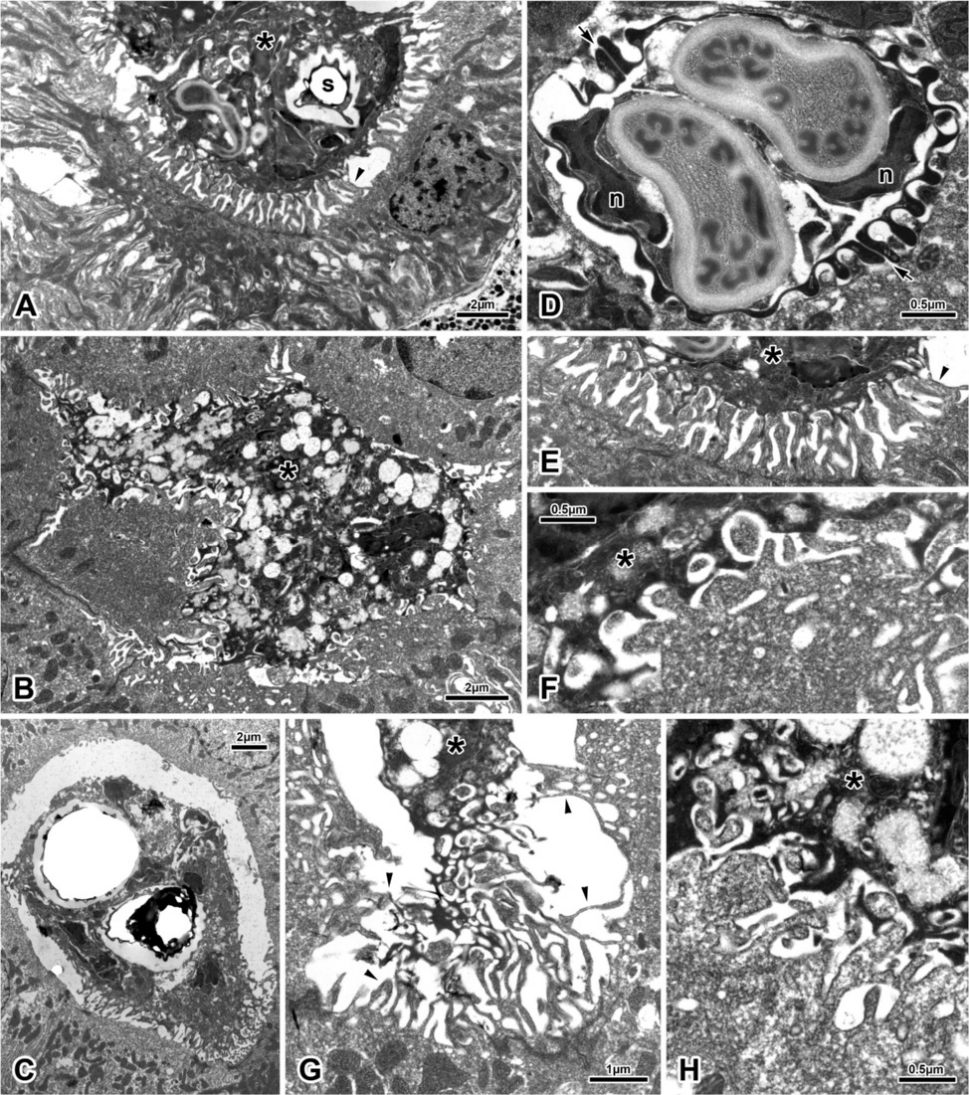
Ultrastructure of *Hoferellus anurae* stages (TEM). **a**-**c** Plasmodia, **d** spore and **e**–**h** pathological alteration of host tubular microvillar zone at the host-parasite interface. Asterisk = plasmodium, *n* = nucleus of capsulogenic cell, s = spore, small arrow = sutural ridge, small arrowhead = altered microvilli. E and F are in the same scale

Apart from mitochondria, various vesicles, spores and electron-dense generative cells, which were often located at the trophozoite periphery, no ultrastructural details could be recognized as a result of sub-standard processing of samples for TEM. Trophozoites filling tubular lumina to various degrees (Fig. 6a), sometimes attached to all surrounding epithelial cells (Fig. 6b), always possessing conspicuous villosities at host-parasite interface (see following section). Spores apparently not formed in pairs, possessing 10–13 longitudinal ridges per valve (Fig. 6d).

#### Mode of attachment to host epithelium

Trophozoites invariably possessing conspicuous villosities and protrusions wherever they are in contact with microvillar surface of the host tubular epithelium. These represent long, often very fine, protrusions of trophozoite deeply inserted into tubular microvillar zone, as well as invaginations of trophozoite surface (Figs 6e–h). The host-parasite interface is formed by a complex mass of intermingling host microvillar zone and parasite surface of spongious appearance. Microvillar zone of infected tubules seemed pathologically affected by the action of plasmodia in all sections. Although microvillar zone of some tubules was still clearly discernible, the tubules were always irregularly fused and deformed (Figs 6a, e). More often, the tubular zone completely lost its integrity, being composed of irregular protrusions and invaginations intermingled with plasmodial surface folds and projections (Figs 6f–h). In many cases, the close connection between the parasite and the host epithelium was restricted to limited area(s) of plasmodial surface. In such situations, the rest of the plasmodium possessed only little projections and corresponding luminal surface of epithelial cells was rather smooth with only faint remnants of former microvillar zone (Fig 6c). In other cases, plasmodial projections were deeply embedded between altered, apparently swollen tubular epithelial cells (Fig 6b).

### Taxonomic summary

Type host: *Afrixalus dorsalis* Peters, 1875 (Anura: Hyperoliidae).

Type locality: Nigeria (no exact location provided in original description).

Other records – all from representatives of Hyperoliidae: *Hyperolius concolor* Hallowell, 1844 (Ghana [47]), *Hyperolius* sp. (Tanzania [47]), *H. kivuensis* (Kenya, this study), *H. viridiflavus* (Kenya, this study).

#### Remarks

Although stated in the original description by Mutschmann [47], posterior spore filaments are not apparent in his micrographs. After thorough examination of numerous fresh mounts and unpublished original photographic documentation from material on which the original description was based (Mutschmann, pers. comm.), we believe the posterior spore filaments, generally distinct and numerous in other nominal *Hoferellus* spp., including *H. gilsoni*, are absent in *H. anurae*. In *Hoferellus gnathonemi* sp. n., the posterior filaments seem to be absent too, but the two posterior projections are markedly longer in this species (compare Figs 3 and 4). Thus, the presence of only two small projections in *H. anurae*, is considered herein a species diagnostic feature. Although presence of very fine and short posterior filaments cannot be ruled out based on light microscopical observations, no such structures were detected in our TEM preparations. According to close phylogenetic and biogeographical relationships of vertebrate hosts, we consider our samples conspecific with *H. anurae* of Mutschmann [47], which is likely a specific parasite of renal tubules of hyperoliid anurans endemic to Sub-Saharan Africa. Thus far, *H. anurae* is documented from West-Central and East Africa, but is to be expected throughout the distribution range of hyperoliid frogs.

### rDNA sequences and phylogenetic results

Partial SSU rDNA sequences obtained in this study are listed in Table 1. The SSU rDNA sequence from *C. auratus* was identical with the published partial sequence of *H. carassii* (GenBank Acc. No. JQ801547, also from Czech Republic) and identical with the SSU rDNA sequence from *C. gibelio* in the present study. All SSU rDNA sequences from *Hoferellus* spp. in *C. carpio* were identical except one isolate from a single common carp from Chřešťovice, which showed 2.6 % sequence divergence and will hereafter be addressed as *Hoferellus* sp. ex *C. carpio*. Sequence variability among *H. carassii* and other *Hoferellus* isolates from carp ranged from 7.4–8.0 %. Interspecific SSU sequence variability revealed very high sequence variability (>20 % variability) between *Hoferellus* spp. in cyprinids and other *Hoferellus*: *H. anurae*, *H. gnathonemi* sp. n. (present study) and *H. gilsoni* (GenBank Acc. No. AJ582062) from *A. anguilla*.

**Fig. 7:**
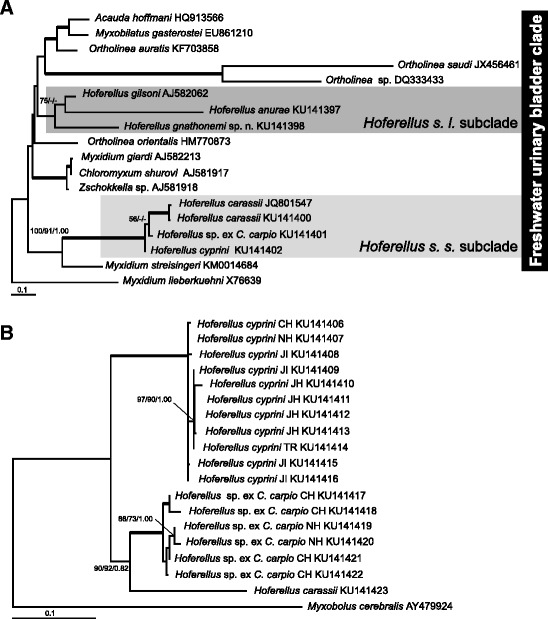
Maximum likelihood trees. Numbers at the nodes represent bootstrap values and Bayesian posterior probability (ML/MP/BI) gaining more than 50 % support (ML and MP) and 0.5 posterior probability (BI). Bold branches lead to a node with a bootstrap support of P ≥ 95 and a Bayesian posterior probability of P ≥ 0.97. Scale bar is given under the tree. **a** Phylogenetic position of *Hoferellus* spp. within the freshwater urinary bladder clade based on SSU rDNA; **b** Phylogenetic reconstruction of *Hoferellus s. s.* relationships based on the basis of ITS rDNA sequences (ITS1, 5.8S, ITS2) with *Myxobolus cerebralis* set as an outgroup. Localities: CH, Chřeštovice, JI Jihlava; JH, Jindřichův Hradec; NH, Nové Hrady; TR, Třeboň

All the newly obtained SSU rDNA sequences cluster within the freshwater urinary bladder clade (Fig. 7a). The genus *Hoferellus* is polyphyletic forming two separate sublineages. Species infecting cyprinids formed a well-supported subclade, hereinafter named as *Hoferellus sensu stricto* (*Hoferellus s. s*.) defined by inclusion of the type species *H. cyprini*, followed by *H. carassii*, and *Hoferellus* sp. ex *C. carpio*. The closest relative of the *Hoferellus s. s*. subclade is *Myxidium streisingeri* with high nodal support. *H. gilsoni* and the other two *Hoferellus* spp., sequenced in this study *i.e. H. anurae* and *H. gnathonemi* sp. n. formed a separate subclade with weak nodal support, hereinafter *Hoferellus sensu lato* (*Hoferellus s. l*.). The close relationship of *H. anurae* with *H. gnathonemi* and *H. gilsoni* was not stable and *H. anurae* showed an affinity to *Ortholinea saudii* and *Ortholinea* sp. from marine fish *Siganus rivulatus* Forsskål & Niebuhr, 1775 in BI analysis (data not shown).

**Fig. 8:**
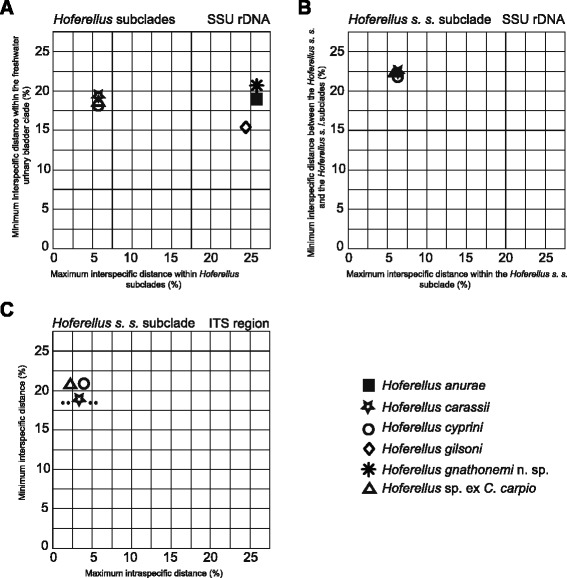
Graphic interpretation of intraspecific and interspecific distances within *Hoferellus* spp. **a** SSU rDNA minimum interspecific distances (dissimilarities) between hoferellids and other members of the freshwater urinary bladder clade plotted against maximum interspecific distance within *Hoferellus s. s.* species and within *Hoferellus s. l.* species; **b** SSU rDNA minimum interspecific distances between species of *Hoferellus s. s.* subclade and species of *Hoferellus s. l.* subclade plotted against maximum interspecific distance within *Hoferellus s. s.* species; **c** ITS minimum interspecific distances among *Hoferellus s. s.* species plotted against their maximum intraspecific distances

The analysis of interspecific SSU sequence distances revealed high minimum sequence dissimilarity (>20 %) between members of *Hoferellus s. s*. and *Hoferellus s. l*. (Fig. 8a, y-axis), whereas genetic distances within the *Hoferellus s. s*. subclade showed 6.6 % maximum interspecific dissimilarity (Fig. 8b, x-axis) in contrast to 26.3 % of maximum interspecific dissimilarity within members of the *Hoferellus s. l*. subclade (Fig. 8a, x-axis). All *Hoferellus* spp. revealed similar minimum sequence dissimilarity to other members of freshwater urinary clade (19.8–21.9 %) except of *H. gilsoni* with the minimum sequence dissimilarity of 15.4 % (Fig. 8a, y-axis). Comparison of clones of the ITS region revealed up to 4 % of maximum intraspecific sequence dissimilarity in sequences of *Hoferellus s. s*. and approximately 20 % of minimum sequence dissimilarity between the three *Hoferellus s. s*. species (Fig. 8c).

*Hoferellus* spp. SSU sequences presented inserts in the variable regions. Two inserts of 15 bp and 45 bp respectively in the V2 region were shared by members of *Hoferellus s. s*. subclade. No inserts were shared by members of *Hoferellus s. l*. subclade, except for an insert of 25 bp present in *H. gnathonemi*. Inserts in V4 regions are 27 bp long only in members of the *Hoferellus s. s.* subclade. *M. streisingeri* has long inserts in V2, V4 and V7 regions with the longest insert in V4 region (150 bp).

The ITS rDNA sequences of *Hoferellus s. s.* from cyprinids were obtained by sequencing of clones from fish obtained from different localities (Table 1): All clones from different goldfish (one site, Jihlava) were identical. Two groups of cloned sequences were observed in carp: 4 clones from Jihlava, 1 from Třeboň, 1 from Chřešťovice and 4 from Jindřichův Hradec (group 1: 0–15.1 % intragroup sequence divergence; *H. cyprini*) and 4 clones from Chřešťovice and 2 from Nové Hrady (group 2: 1–18 % intragroup sequence divergence; *Hoferellus* sp.). Sequence divergence between the two groups of clones from carp ranged from 18.2–21.9 %, which was very similar to the variability observed between *H. carassii* and all carp clones (22.5–24.5 %).

Phylogeny of the cloned sequences of the ITS rDNA region of *Hoferellus* spp. from common carp and goldfish showed three well-defined branches (Fig. 7b). An individual sample from common carp from Třeboň, K102 (Acc. No. KU141414) provided a single sequence, and was associated with typical *H. cyprini* spores as described in the present work. All the sequences of clones clustering with this isolate were considered conspecific (Fig. 7b). The other sequences from common carp that did not cluster with this isolate were considered a different species, *Hoferellus* sp. ex *C. carpio* (K41 clones Acc. No. KU141406, which were obtained from the same host as the SSU sequence of *Hoferellus* sp. ex *C. carpio*, Acc. No. KU141401). Based on the ITS region, intraspecific variability was revealed for *H. cyprini* and *Hoferellus* sp. ex *C. carpio*, but not for *H. carassii* for which only one sequence was obtained from different clones analysed, however, they all came from the same site. The results from the phylogenetic analyses of the ITS rDNA region provided further support to the SSU rDNA based phylogeny revealing three distinctive separate species in *Hoferellus s. s.* subclade.

### Single and duplex PCR detection of *Hoferellus* spp. in cyprinids

The results of the combination of PCR assays designed to differentiate between the three species/genotypes of *Hoferellus* in cyprinids are summarized in Table 1. The assay showed that goldfish and Prussian carp were exclusively infected with *H. carassii* whereas common carp was infected with *H. cyprini* and *Hoferellus* sp. ex *C. carpio*. Both *H. cyprini* and *Hoferellus* sp. ex *C. carpio* were detected in all localities (except for Bavorov) and higher prevalence was generally observed for *H. cyprini* (26.7–60 %) than for the other genotype (12.8–50 %). Mixed infections of *H. cyprini* and *Hoferellus* sp. ex *C. carpio* in common carp were detected only molecularly, with prevalences ranging between 13.3–25 % at a range of localities (see Table 1).

PCR detection of *H. carassii* in *C. auratus* confirmed the results observed microscopically with higher prevalence in the ureters and urinary bladders than in the kidneys (Table 1). Simultaneous presence of *H. carassii* in kidney and urinary ducts was detected only in 3 goldfish (2 from Jihlava, in June and 1 from Chřešťovice, in September). *H. carassii* infections in *C. gibelio* (June and August 2012) were only detected in ureters and urinary bladders, however, all kidneys were PCR negative. *H. cyprini* was detected in similar prevalence in kidney and urinary ducts/bladders (40.9 % 9/22 *vs* 44.8 % 13/29), whereas *Hoferellus* sp. ex *C. carpio* molecular prevalence in urinary ducts/bladders (24.2 % 8/33) was higher than in kidneys (12.9 % 8/62).

## Discussion

Almost all year round different myxozoan developmental stages were detected in the lumina of the renal tubules of cyprinid fishes in Czech ponds. A high diversity of myxozoan species infecting the urinary system is known from cyprinid fishes, including species of the genera *Sphaerospora*, *Buddenbrockia* or *Myxidium* [48, 49]. The lack of spores makes *Hoferellus* infections difficult to discriminate from other myxozoans, hence, molecular methods are essential for diagnosis, but also to determine true prevalences in case of low infection levels, and to reveal the true diversity of *Hoferellus* spp. in selected West Paleartic cyprinids.

### *Hoferellus* as a polyphyletic genus

The present study provides 4 new SSU rDNA sequences for members of the genus *Hoferellus*, including the type species *H. cyprini*. Phylogenetic analyses of 6 genotypes of nominal species demonstrate that *Hoferellus* is yet another polyphyletic myxozoan genus. Although most relationships within the freshwater urinary clade, which all *Hoferellus* spp. belong to, are characterised by low bootstrap support, the polyphyly of hoferellids is confirmed by a strongly supported sister relationship of *M. streisingeri* with the *Hoferellus s. s.* subclade. Hence, the *Hoferellus* morphotype seems to have evolved more than once in the freshwater clade.

High maximum interspecific dissimilarity within members of *Hoferellus s. l.* is caused due to the divergent SSU rDNA sequence of *H. anurae*. This species is characterised by a very long-branch in the phylogenetic tree, which could be explained by the longer independent evolution of *H. anurae* or by the accelerated evolution of this species, potentially due to its occurrence in amphibians rather than fish. Similar long-branch character of SSU is seen in two closely related *Ortholinea* species. Long branching species can be problematic for reliable phylogenetic reconstruction since their position can be affected by long-branch attraction [50]. Due to the weak support for the position of *Hoferellus s. l*. their exact position is not determined until other close relatives are found. *Hoferellus s. l*. cluster closely with several marine *Ortholinea* species, which would suggest several evolutionary switches between marine and freshwater environments. The most parsimonious explanation of this phylogeny would be a more basal position of *Hoferellus s. l*. and a closer relationship with marine *Ortholinea* spp.

### Host specificity of *Hoferellus s. s.* from West Palearctic cyprinids

Our molecular identification and PCR screening results of *Hoferellus* spp. in cyprinid fishes of Czech ponds clarifies the much-discussed identity of these species [12, 13, 17] (see Tables 3 and 4). The type species, *H. cyprini*, and the second genotype detected, appeared to be specific for common carp, *C. carpio,* and they did not infect goldfish or Prussian carp. *H. cyprini* is slightly more prevalent than *Hoferellus* sp. ex *C. carpio*, although mixed infections are common. Despite the large sample size, we observed spores of only a single *Hoferellus* species in common carp. Sequences from these always included the genotype here identified with *H. cyprini*. Only by molecular tools was it possible to detect both species in carp, revealing that *Hoferellus* sp. ex *C. carpio* more frequently infects the ureters and urinary bladders, whereas *H. cyprini* was detected all along the excretory system, suggesting that these taxa show sympatric speciation by exploiting slightly different host habitats, and they likely speciated relatively recently.

A different scenario was observed in *H. carassii*. This species was originally described from Prussian carp by Akhmerov [22] and reported from different hosts according to morphological identification (see Table 3). We confirmed by species-specific duplex PCR that *H. carassii* infects at least two different host species of the genus *Carassius*, *C. auratus* and *C. gibelio*, but it does not infect common carp, *C. carpio*. A relaxed host-specificity for the two closely-related *Carassius* species [51] that were considered subspecies before, was also observed in other, generally highly host-specific genera, such as *Sphaerospora* [48]. Furthermore, SPF (specific pathogen free) common carp exposed to the actinosporean stage of *H. carassii*, produced by the invertebrate host did not result in infection under experimental conditions [25]. *H. carassii* seems to develop differently in the two *Carassius* hosts: Previously, *H. carassii* in *C. auratus* was found in kidney tubules and urinary ducts, whereas in *C. gibelio* it infects only the urinary bladder [19]. This agrees with our results of both microscopy and PCR in *C. gibelio*. This different infection pattern may indicate host-dependent variability in habitat selection of *H. carassii*, potentially reflecting the onset of a speciation process.

### Pre-sporogonic development of *Hoferellus s. s.* and the identity of enigmatic intracellular stages

An annual life cycle was assumed for *H. cyprini* in common carp, with early intracellular stages in the tubular epithelium during summer, mature intracellular stages in the syncytium containing up to quaternary cells in autumn and winter, that are released gradually into the lumen from winter to spring, producing mature spores in spring [19, 27]. Similarly, an annual cycle was described in *H. carassii* from goldfish: intracellular stages in October-December, luminal stages in January-February and spores in April [17, 20]. In the present study, common carp and goldfish were sampled from February to November. Although abundant coelozoic (luminal) stages were found for both species throughout the sampling period, no intracellular stages were detected at any time or locality in the present study. A possible explanation therefore may be a low infection intensity in the fish analysed in the present study, potentially leading to a low number and thus great difficulty in detecting these stages. Prevalence data for *Hoferellus* intracellular stages are rare but existing ones are relatively low: 1/12 common carp, January [52], 1/42 gibel carp, October and 3/12 goldfish, April-November [19]. Intracellular myxozoan stages in renal tubules epithelia in carp and coho salmon were suggested to be abortive extrasporogonic developmental stages of other myxozoan species, *e.g. Sphaerospora* or *Myxidium*, which stages seemed to degrade, instead of following through to sporogony [16, 29]. In a similar manner, *Hoferellus* sp. ex *C. carpio* may be unable to complete its development and produce spores, as they were never detected microscopically. It may well be a related species that sporulate in a different, likely related host, and hence only initial stages of development can be found in carp. For example, *Myxobilatus gasterostei* (Parisi, 1912), a species with intraepithelial development in the kidney tubules of sticklebacks, was reported in the blood of *C. carpio* and *C. auratus* as an alien species that does not form spores in these hosts [6, 48]. Alternatively, intracellular stages might also belong to a transient developmental stage of the malacosporean *Buddenbrockia* sp. 2, a common species in the kidney of common carp and goldfish [48, 49, 53]. In the present study, intracellular presporogonic stages with several secondary cells were described in frog kidneys. Unfortunately, no molecular proof could be provided in this study and they were putatively assigned to *H. anurae,* a member of *Hoferellus s. l*. Further molecular studies are needed to identify the species to which the enigmatic intracellular stages belong to.

### Pathogenicity of nominal *Hoferellus* spp

*H. carassii* is suggested to be the causative agent of KED in goldfish [19, 20]. The renal damage related to KED is caused by *H. carassii* stages invading the epithelial cells of the renal tubules, with disease symptoms only observed in pet fish populations kept in small ponds, but not in natural fish populations [19]. Despite the high prevalence of infection in goldfish kept in small ponds for sale as ornamental fish, no mortalities or signs of disease were observed in the present study or in a previous study [25]. A possible explanation could be the mild infections in the goldfish from our study, which also disabled the detection of the intracellular stages.

In 2004, Mutschmann described ‘frog kidney enlargement disease’ in hyperoliid anurans from the pet trade and identified its causative agent as *H. anurae*, which he formally described and named in the same study. Although we did not detect any gross renal pathology even in heavily infected (but otherwise obviously healthy) frogs from a wild population, we detected pathological changes on an ultrastructural level. The pronounced deterioration of the microvillar zone and swelling of epithelial cells of infected renal tubules might imply that *H. anurae* has a potential to cause disease under certain conditions, such as host immunosuppression or heavy infections. This might explain the apparent absence of the disease in wild frog populations in contrast to the severe impact of the infection on frogs likely subjected to suboptimal and stressful conditions associated with the pet trade.

### *Hoferellus s. l.* diversity and host-parasite interactions

Morphologically, members of *Hoferellus s. l.* have slightly different spore shapes. *H. gilsoni* and *H. gnathonemi* parasitize teleost fishes and have round or subspherical spores produced in pairs, whereas *H. anurae* infect frogs, possess pyramidal or miter-like spores which are flat at the posterior end and do not develop in pairs. *H. gilsoni* and *H. anurae* seem to share a rather unique mode of attachment to the epithelia of its host, the urinary bladder [30] and kidney tubules (present study), respectively.

Phylogenetically, all three species, *H. gilsoni*, *H. anurae* and *H. gnathonemi* clearly clustered separately from *Hoferellus s. s.* However, the phylogenetic relationship between them was not resolved with the presently available sequence data: our SSU rDNA phylogenetic results showed no clear clustering of the three species. Therefore, we suggest their temporal classification as *Hoferellus s. l.* before more morphological and molecular information is available for substantiated taxonomical rearrangements.

### Taxonomy and species currently assigned to genus *Hoferellus* Berg, 1898

The genus *Hoferellus* was originally described by Doflein in 1898 [43] as *Hoferia*, with *Hoferia cyprini* as type species. However, the name *Hoferia,* had been assigned to an extinct genus of molluscs by Bittner in 1894 [54]. The genus was then renamed as *Hoferellus* (*nov. nom. pro.*) by Berg in 1898 [8]. The genus *Mitraspora* Fujita, 1912 is a further synonym of *Hoferellus* according to Lom 1986 [12]. The establishment of *Mitraspora* was probably based on a different suture position on the spores [13]. In the original species description of *H. cyprini* and *H. carassii* the suture was wrongly described as in the same plane as that of the polar capsules. *Mitraspora* was established with *Mitraspora cyprini* (Fujita, 1912) as type species, to include species that had resemblance with *Hoferellus* species but have a suture perpendicular to the plane of the polar capsules. *Mitraspora* was synonymized with *Sphaerospora* by Shulman (1966) [10] probably because in sphaerosporids the suture position is also perpendicular to the polar capsules. *Mitraspora* was considered again as a separate genus by Lom & Noble (1984) [11], assigning *H. carassii* as type species of the genus *Hoferellus* and *M. cyprini* as type species of *Mitraspora*. Finally, the genus *Mitraspora* was considered a junior synonym of *Hoferellus* [12, 13, 27] with *H. cyprini* as the type species.

All *Hoferellus* spp. (syn. *Mitraspora*) described to date and for which DNA sequence data is missing, should be addressed as *Hoferellus incertae sedis* until SSU rDNA sequences are obtained and they can be ascribed to *Hoferellus s. s.* or *s. l. H. anurae*, *H. gilsoni* and *H. gnathonemi* will remain *Hoferellus s. l.,* although they may resolve in new genera, at a later stage.

According to the review by Lom & Dyková (2006) [5], there are 25 species in the genus *Hoferellus*. After this review, only two species, *Hoferellus jurachni* Moshu & Trombitsky, 2006 and *Hoferellus pulvinatus* Baska *et al*., 2009 were described and another species was moved to the genus *Acauda* [55]. Although *Mitraspora* was synonymized with *Hoferellus* in 1986, species had still been ascribed to the genus *Mitraspora*. Two species were named with the same specific name, *i.e. Hoferellus sinensis* Li & Nie, 1965 and *Mitraspora sinensis* Li & Nie, 1965 from different hosts [26, 56].

#### Summary

##### *- Hoferellus s. s*.

*Hoferellus cyprini* (Doflein, 1898)

*Hoferellus carassii* Akhmerov, 1960

(*Hoferellus* sp. ex *C. carpio*)

***- Hoferellus s. l.***

*Hoferellus gilsoni* (Debaisieux, 1925)

*Hoferellus anurae* Mutschmann, 2004

*Hoferellus gnathonemi* sp. n.

***- Hoferellus incertae sedis***

*Hoferellus caudatus* (Parisi, 1910), *Hoferellus plecoglossi* (Fujita, 1927), *Hoferellus caspialosae* (Dogiel & Bychovsky, 1939), *Hoferellus dubinini* (Shulman, 1962), *Hoferellus donecii* (Gazimagomedov, 1970), *Hoferellus sichuanensis* Ma, Dong & Wang, 1982, *Hoferellus minuta* Nie & Li, 1992, *Hoferellus anguilli* Hsieh & Gong, 1993, *Hoferellus orientalis* Zao & Ma, 1997, *Hoferellus coreosiniperca* Xiao-Chongxue & Feng, 1997, *Hoferellus hupehensis* Chen, 1998, *Hoferellus wuchangensis* Chen, 1998, *Hoferellus hunanensis* Chen, 1998, *Hoferellus liocasis* Chen, 1998, *Hoferellus glyptothoraxi* Ma, 1998, *Hoferellus changkiangensis* Ma, 1998, *Hoferellus yiduensis* Gong, Lu & Wang, 2004, *Hoferellus jurachni* Moshu & Trombitsky, 2006, *Hoferellus pulvinatus* Baska et al., 2009

Others

*Hoferellus* sp. Fantham, 1919

**Emendation of the genus*Hoferellus*Berg**, **1898** after Lom & Dyková, 2006 [5].

syn. *Hoferia* Doflein, 1898 (hom. *Hoferia* Bittner, 1894), syn. *Mitraspora* Fujita, 1912.

Myxospores pointed in valvular view, miter/bullet-shaped, longitudinal ridges along surface of valves, some of them continuing into caudal filaments at posterior end. Suture perpendicular to polar capsule plane. Polar capsules pyriform, sporoplasm binucleate, trophozoites polysporic. Complete life cycle revealed, with actinospores known from oligochaetes of the Naidinae. Coelozoic in the urinary system of freshwater fishes. Some with conspicuous intracellular development. Number of species: 27.

Type species: *Hoferellus cyprini* (Doflein, [43]).

Type host: *Cyprinus carpio*

### Remarks

*H. cyprini* actinospores were described as an aurantiactinomyxon released from the oligochaete *Nais* sp. [57]. No consensus exists about the actinosporean stage of the other species, *H. carassii*: *H. carassii* aurantiactinomyxon type spores released from tubificids [58] and *Nais* sp. [25] and/or neoactinomyxon type spores from the oligochaete *Branchiura sowerbyi* [23]. All hoferellid life cycles were elucidated based on experimental infections, and, to date, none of the life cycle stages from annelids and fish have been related to each other by DNA sequencing.

## Conclusions

This study aimed to provide a synthesis of presently described and known taxa compromising the myxozoan genus *Hoferellus* and provides an initial set of SSU rDNA sequences of 5 representatives of this genus. We determined a polyphyletic nature of *Hoferellus*, with two sublineages (*Hoferellus s. s.*, which hosts the type species, and *Hoferellus s. l.*), which are indistinguishable with regard to their spore morphology. Molecular and phylogenetic tools allowed us to resolve cryptic species *of Hoferellus s. s.* and their host specificity in Western Palearctic cyprinids, probably a result of a recent speciation processes in the urinary tract of common carp in the Czech Republic. We suggest the *Hoferellus s. l.* species to remain members of the genus until their phylogenetic position and relationship with other taxa is further resolved. We demonstrate that molecular methods are inevitable to better understand the relationships between hoferellids and to define their taxonomic status.
